# Manipulation of Phytohormone Pathways by Effectors of Filamentous Plant Pathogens

**DOI:** 10.3389/fpls.2019.00822

**Published:** 2019-06-26

**Authors:** Xiaowei Han, Regine Kahmann

**Affiliations:** Department of Organismic Interactions, Max Planck Institute for Terrestrial Microbiology, Marburg, Germany

**Keywords:** filamentous plant pathogen, effector, virulence, phytohormone, plant defense signaling

## Abstract

Phytohormones regulate a large variety of physiological processes in plants. In addition, salicylic acid (SA), jasmonic acid (JA), and ethylene (ET) are responsible for primary defense responses against abiotic and biotic stresses, while plant growth regulators, such as auxins, brassinosteroids (BRs), cytokinins (CKs), abscisic acid (ABA), and gibberellins (GAs), also contribute to plant immunity. To successfully colonize plants, filamentous pathogens like fungi and oomycetes have evolved diverse strategies to interfere with phytohormone pathways with the help of secreted effectors. These include proteins, toxins, polysaccharides as well as phytohormones or phytohormone mimics. Such pathogen effectors manipulate phytohormone pathways by directly altering hormone levels, by interfering with phytohormone biosynthesis, or by altering or blocking important components of phytohormone signaling pathways. In this review, we outline the various strategies used by filamentous phytopathogens to manipulate phytohormone pathways to cause disease.

## Introduction

Filamentous plant pathogens, like fungi and oomycetes, cause severe crop yield losses annually (Fisher et al., 2012). To protect themselves against pathogens, plants have evolved a multilayered defense network (Jones and Dangl, 2006). This immune system is activated when membrane localized pattern recognition receptors (PRRs) recognize microbe-associated molecular patterns (MAMPs) or host-derived damage-associated molecular patterns (DAMPs), leading to pattern triggered immunity (PTI) (Couto and Zipfel, 2016). The activation of PTI results in an array of cellular responses, including the generation of extracellular reactive oxygen species (ROS), cytosolic ion-flux changes, calcium-dependent or mitogen-activated protein kinase cascade activation, reinforcement of physical barriers, and the production of numerous defense-related molecules (Macho and Zipfel, 2014; Couto and Zipfel, 2016). In these complex immune responses, phytohormones play pivotal regulatory roles. Classical defense phytohormones are salicylic acid (SA), jasmonic acid (JA), and ethylene (ET). More recently, growth-related phytohormones, such as auxins, cytokinins (CKs), brassinosteroids (BRs), abscisic acid (ABA), and gibberellins (GAs) are also shown to modulate plant immune defenses (Pieterse et al., 2009, 2012; Tsuda and Katagiri, 2010; Berens et al., 2017).

Filamentous plant pathogens which can successfully colonize plants secrete an arsenal of effector proteins to interfere with plant defenses and facilitate pathogen colonization (Jones and Dangl, 2006; Dangl et al., 2013; Lo Presti et al., 2015). These effectors are categorized into two groups: apoplastic effectors that reside and function in the apoplast and cytoplasmic effectors that are taken up by plant cells to target various intracellular processes (Kamoun, 2006). In oomycetes, many of the cytoplasmic effectors are so called RxLR and Crinkler (CRN) effectors possessing an N-terminal RxLR motif or LxLFLAK motif, respectively, which are implicated in effector uptake (Jiang et al., 2008; Schornack et al., 2010; McGowan and Fitzpatrick, 2017). Fungal effectors which are taken up by host cells lack such a consensus motif. Given the importance of phytohormone pathways in plant immunity, it is no surprise that filamentous plant pathogens have evolved protein or toxin effectors targeting hormonal pathways. In addition, filamentous plant pathogens can also produce phytohormones and derivatives as host mimicry to manipulate or hijack host hormone homeostasis (Chanclud and Morel, 2016). In this communication, we review recent findings illustrating how this is achieved and discuss how such molecules enhance parasite fitness.

## Effectors Targeting the Salicylic Acid Pathway

The phytohormone SA is a phenolic compound involved in various plant processes including growth, flowering, thermogenesis, senescence, and responses against abiotic and biotic stress (Raskin, 1992; Vlot et al., 2009; Dempsey et al., 2011). SA has been extensively studied for its role in local and systemic acquired resistance (LAR and SAR) against biotrophic and hemibiotrophic pathogens (Malamy et al., 1990; Metraux et al., 1990; Raskin, 1992; Klessig and Malamy, 1994; Glazebrook, 2005; Vlot et al., 2009; Dempsey et al., 2011).

**Figure 1:**
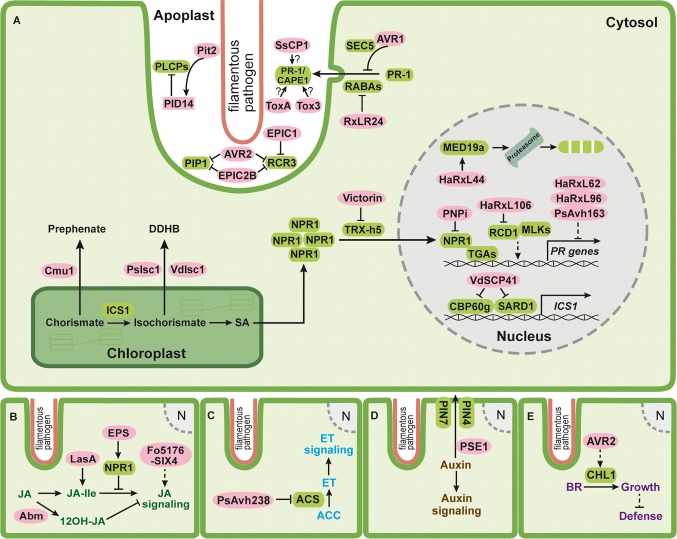
Schematic overview of effectors of filamentous phytopathogens targeting phytohormone pathways. **(A)** SA (salicylic acid) pathway; **(B)** JA (jasmonic acid) pathway; **(C)** ET (ethylene) pathway; **(D)** auxin pathway; and **(E)** BR (brassinosteroid) pathway. Infection structures of filamentous pathogens penetrating a plant cell are lined with salmon color. This structure or specialized feeding structures (not indicated) are the sites for secretion of pathogen effectors. The plant plasma membrane is shown in green, the plant cytosol is shown in light green, the chloroplast is outlined with dark green, and the plant nucleus is shown in gray. In **(A)**, the apoplastic space between pathogen and plant plasma membrane is enlarged. Pathogen effectors residing either in the apoplast or in the plant cytosol are indicated by pink ovals. Plant components targeted by effectors are depicted as rounded green rectangles. Solid lines represent characterized reactions or direct interactions and dashed lines represent indirect interactions. Arrows indicate activation and bar headed lines indicate inhibition. Question marks indicate that the underlying mechanism is not yet clear.

SA is synthesized from chorismate, the end product of the shikimate pathway, *via* two distinct biosynthetic pathways. The phenylalanine ammonia lyase (PAL) pathway starts with the Claisen rearrangement of chorismate to prephenate catalyzed by chorismate mutase, followed by the formation of phenylalanine. Subsequently, PAL catalyzes the conversion of phenylalanine to cinnamate, which can be converted to SA in a series of enzymatic steps (Klessig and Malamy, 1994; Metraux, 2002; Dempsey et al., 2011). In the isochorismate (IC) pathway, chorismate is converted to SA in the chloroplast *via* two reactions catalyzed by isochorismate synthase (ICS) and isochorismate pyruvate lyase (IPL), respectively (Figure 1A; Wildermuth et al., 2001; Strawn et al., 2007; Garcion et al., 2008; Dempsey et al., 2011).

To interfere with SA-mediated defenses, a direct and efficient way is to prevent the formation of SA. This strategy has been exploited by several filamentous plant pathogens. The biotrophic fungus *Ustilago maydis*, which is the causative agent of corn smut disease, secretes an active chorismate mutase Cmu1 converting chorismate to prephenate (Figure 1A; Djamei et al., 2011). Plants infected with *cmu1* deletion mutants displayed significantly higher SA levels, and such mutants were less virulent than wild type strains (Djamei et al., 2011). Cmu1 is a cytoplasmic effector and it is postulated that translocated Cmu1 increases cytosolic chorismate mutase activity and diverts the flow of chorismate into the phenylpropanoid pathway, thus hindering SA biosynthesis and immunity against this biotrophic pathogen (Figure 1A; Djamei et al., 2011). Secreted chorismate mutases are not only found in several other smut fungi but also in the necrotrophic fungus *Sclerotinia sclerotiorum*. This could suggest that lowering SA levels might be a common strategy to suppress host defenses (Djamei et al., 2011; Kabbage et al., 2013; Derbyshire et al., 2017). However, Cmu1 was recently also found to interact with the maize kiwellin *Zm*KWL1 in the apoplast (Han et al., 2019). *Zm*KWL1 was shown to be a defense-related protein, which significantly inhibited the chorismate mutase activity of Cmu1 (Han et al., 2019). The interaction of Cmu1 with *Zm*KWL1 suggests that Cmu1 has an additional role in the apoplast besides its metabolic reprogramming activity. For the secreted chorismate mutase of *S. sclerotiorum*, it still needs to be investigated whether this protein affects SA metabolism or inhibits apoplastic defense responses. An alternative way to lower SA levels is employed by the oomycete pathogen *Phytophthora sojae* and the fungus *Verticillium dahliae*, which secrete isochorismatases PsIsc1 and VdIsc1, respectively, *via* an unconventional route (Liu et al., 2014). PsIsc1 was shown to function inside plant cells (Figure 1A; Liu et al., 2014). Isochorismatases convert isochorismate to 2,3-dihydro-2,3-dihydroxybenzoate (DDHB) and pyruvate, making isochorismate unavailable for SA biosynthesis. Silencing of *PsIsc1* in *P. sojae* or inactivation of *VdIsc1* in *V. dahliae* increased SA levels in infected plant tissue and led to the induction of the SA marker gene *pathogenesis-related protein 1 (PR-1)* (Liu et al., 2014). Interestingly, some fungi also produce salicylate hydroxylases that degrade SA to catechol in the fungal cytosol, which could potentially contribute to lowering SA levels in infected tissue. However, so far, salicylate hydroxylases are not yet implicated in virulence.

SA biosynthesis is tightly regulated by a complex transcriptional network (Vlot et al., 2009; Dempsey et al., 2011). Two closely related transcription factors calmodulin-binding protein 60 g (CBP60g) and SAR deficient1 (SARD1) positively regulate the SA-induced defense response through binding to promoter region of the SA biosynthetic gene *ICS1* (Figure 1A; Wang et al., 2009, 2011a; Sun et al., 2015, 2018). *cbp60g/sard1* double mutants of *Arabidopsis thaliana* were more susceptible to *V. dahliae* infection, illustrating that CBP60g and SARD1 promote immunity against *V. dahliae* (Qin et al., 2018). The nuclear effector VdSCP41 of *V. dahliae* was recently shown to target *A. thaliana* CBP60g and SARD1 (Figure 1A; Qin et al., 2018). Biochemical assays revealed that VdSCP41 bound to the transcription activation domain in the C-terminus of CBP60g, compromising its transcription activity required for the induction of *ICS1* (Figure 1A; Qin et al., 2018). The deletion of *VdSCP41* in *V. dahliae* reduced virulence, whereas *A. thaliana* plants expressing VdSCP41 exhibited compromised PTI-triggered expression of *ICS1* and showed increased disease symptoms after infection with *V. dahliae* (Qin et al., 2018).

Nonexpressor of PR genes1 (NPR1) is the master regulator of SA-mediated plant immune responses (Cao et al., 1994, 1997; Shah et al., 1997; Dong, 2004; Wang et al., 2006). In uninfected plants, NPR1 oligomers reside in the cytosol in an inactive state. SA production in response to pathogen attack leads to NPR1 phosphorylation and subsequent monomerization, allowing its translocation into the nucleus to activate *PR* gene expression (Figure 1A; Kinkema et al., 2000; Mou et al., 2003; Lee et al., 2015). NPR1 regulates the expression of *PR* genes through interaction with several TGA transcription factors (Figure 1A; Despres et al., 2000; Dong, 2004; Kesarwani et al., 2007; Fu and Dong, 2013). Due to its essential role in plant immunity, NPR1 presents an interesting effector target to subvert SA-mediated defenses (Lorang et al., 2012; Kazan and Lyons, 2014).

A yeast two-hybrid screen with a library from wheat infected by the yellow stripe rust *Puccinia striiformis* f. sp. *tritici* identified a conserved rust protein PNPi (for *Puccinia* NPR1 interactor) as an NPR1 interaction partner (Figure 1A; Wang et al., 2016). PNPi compromised the interaction between NPR1 and TGA2.2 in a yeast three-hybrid assay (Figure 1A; Wang et al., 2016). Overexpression of PNPi in barley reduced the expression of several *PR* genes normally induced upon biotic stress, suggesting that PNPi might reduce *PR* gene expression *via* blocking the binding of NPR1 to TGA transcription factors also *in vivo* (Wang et al., 2016). The necrotrophic fungus *Cochliobolus victoriae* causes Victoria blight on oat and *A. thaliana* by secreting the cyclic peptide toxin effector victorin (Lorang et al., 2004, 2007). Victorin sensitivity in *A. thaliana* requires both the locus orchestrating victorin effects1 (LOV1) belonging to the nucleotide-binding leucine rich repeat (NLR) protein family and the defense-associated thioredoxin TRX-h5 (Lorang et al., 2007; Sweat and Wolpert, 2007). TRX-h5 is upregulated in response to pathogen challenge and catalyzes the critical conformational change of NPR1 from the oligomeric to monomeric state (Figure 1A; Laloi et al., 2004; Tada et al., 2008). In *A. thaliana* plants lacking *LOV1*, victorin binds to the active site of TRX-h5 and inhibits its activity, thus blocking monomerization of NPR1 and subsequent SA-mediated defense responses (Figure 1A; Lorang et al., 2012). When LOV1 is present, it acts as the guard of TRX-h5. The binding of victorin to TRX-h5 activates LOV1 and leads to hypersensitive response (HR)-like cell death, which favors the necrotrophic lifestyle of *C. victoriae* (Lorang et al., 2012). This illustrates that *C. victoriae* hijacks the guard function of LOV1 to evoke cell death and facilitate necrotrophic development.

*PR-1* gene expression has been extensively employed as an SA marker due to its strong induction during SA-mediated plant immune responses (Lotan et al., 1989; Van Loon and Van Strien, 1999; Dong, 2004). PR-1 proteins are delivered to the apoplastic space and their successful secretion is prerequisite for their functions (Carr et al., 1987; Wang et al., 2005). *PR-1* overexpression as well as *in vitro* studies using spore germination or infection structure differentiation as readouts indicated that PR-1 proteins might show antimicrobial activity (Alexander et al., 1993; Niderman et al., 1995; Rauscher et al., 1999; Sarowar et al., 2005; Kiba et al., 2007). However, very high concentrations were needed to observe such an activity, putting into question the biological relevance of such observations (Breen et al., 2017). Recently, PR-1 was shown to bind sterols and inhibit the growth of *Phytophthora brassicae*, whereas no effect was seen on growth of the fungal species *Aspergillus niger* and *Botrytis cinerea* with the same treatment (Gamir et al., 2017). The inhibition of *P. brassicae* by purified PR-1 protein P14c from tomato could be alleviated when cholesterol was added, suggesting a link between sterol-binding activity and growth inhibition (Gamir et al., 2017). These authors speculate that the selective growth inhibition of the oomycete by P14c may result from the sterol auxotrophy of *P. brassicae*, i.e. *P. brassicae* relies on environmental sterols, and P14c may deplete this supply by binding sterols. The RxLR effector RxLR24 from *P. brassicae* binds several *A. thaliana* RABA GTPases that are required for vesicle-mediated secretion of proteins (Figure 1A; Tomczynska et al., 2018). This inhibited the secretion of PR-1 proteins and presumably other defense proteins, in line with the need to reduce PR-1 levels in the apoplast for disease development of this hemibiotrophic oomycete. A similar situation exists in the potato blight oomycete *Phytophthora infestans*. This hemibiotroph secretes the RxLR effector AVR1 which interacts with and stabilizes Sec5, a subunit of exocyst complex (Figure 1A; Du et al., 2015). Since the secretion of PR-1 requires Sec5, the authors speculate that a stabilized exocyst by AVR1 may block focal secretion of PR-1 and other defense compounds (Figure 1A; Du et al., 2015, 2018). While the suppression of PR-1 levels by oomycete pathogen effectors is in line with their sterol auxotrophy and the sterol-binding activity of PR-1, it is presently not evident why the necrotrophic fungus *S. sclerotiorum*, a sterol prototroph, should secrete the cerato-platanin-like SsCP1 effector that directly interacts with *A. thaliana* PR-1 in the apoplast (Figure 1A; Yang et al., 2018b). ScCP1 also promotes virulence, induces necrosis-like cell death at high concentrations, and activates the SA pathway (Yang et al., 2018b). In addition, the necrotrophic wheat pathogen *Parastagonospora nodorum* secretes two effectors ToxA and Tox3 which target certain PR-1 isoforms including PR-1-5 (Figure 1A; Lu et al., 2014; Breen et al., 2016). PR-1-5 enhanced the necrosis inducing ability of purified ToxA on wheat leaves harboring the toxin sensitivity gene *Tsn1* (Lu et al., 2014). In later studies, CAP-derived peptides (CAPE) were detected in some PR-1 proteins (Chen et al., 2014; Breen et al., 2017). CAPE1 peptides can be proteolytically liberated from PR-1b after wounding, classifying them as DAMPs (Chen et al., 2014). Infiltration with CAPE1 peptides enhanced wheat cell death caused by Tox3 in a wheat line carrying *Snn3* (Breen et al., 2016). It is therefore likely that the positive biological function of PR-1 proteins toward filamentous pathogens might be connected to the liberation of defense signaling CAPE peptides.

During SA-triggered defense responses papain-like cysteine proteases (PLCPs) are secreted to the apoplastic space and play prominent roles in plant immunity against biotrophic and hemibiotrophic filamentous pathogens (Shabab et al., 2008; van der Linde et al., 2012; Misas-Villamil et al., 2016). It was shown that treatment of tomato with the SA analog benzothiadiazole (BTH) specifically induced the transcription of two PLCPs, PIP1, and RCR3 (Shabab et al., 2008). PIP1 and RCR3 are both inhibited by apoplastic effectors from evolutionarily unrelated pathogens, namely AVR2 from the tomato leaf mold fungus *Cladosporium fulvum* and extracellular cystatin-like protease inhibitor 2b (EPIC2B) from the oomycete *P. infestans* (Figure 1A; Tian et al., 2007; Shabab et al., 2008; Song et al., 2009). In addition, RCR3 is also inhibited by EPIC1 from *P. infestans* (Figure 1A; Song et al., 2009). While cystatin-like protease inhibitors like EPIC2B inhibit PLCPs *via* a conserved QxVxG motif, a distinct inhibition mechanism is speculated for AVR2 lacking an QxVxG motif (Shabab et al., 2008; Kaschani et al., 2010; Kaschani and Van der Hoorn, 2011).

Maize PLCPs, such as CP1, CP2, and XCP2, are also activated upon SA treatment as part of the SA-mediated defense response (van der Linde et al., 2012). Recently, an immune signaling peptide, *Zea mays* immune signaling peptide 1 (Zip1), was shown to be released from its propeptide precursor by SA-induced PLCPs and demonstrated to activate downstream SA defense signaling (Ziemann et al., 2018). The maize cystatin CC9 strongly induced upon infection by *U. maydis* was shown to inhibit apoplastic PLCP activity and this promoted *U. maydis* colonization (van der Linde et al., 2012). The virulence-promoting apoplastic effector Pit2 of *U. maydis* inhibits maize PLCPs *via* a novel 14 amino acid long motif (PID14) initially defined by mutational analyses and synthetic peptides (Figure 1A; Doehlemann et al., 2011; Mueller et al., 2013). Recent analyses demonstrated that Pit2 is processed by maize PLCPs, and as a result, a released inhibitory portion inside the PID14 domain remains bound to the PLCP and blocks its activity (Misas Villamil et al., 2019). The *U. maydis* Pit2 effector thus functions as a substrate mimicking molecule. The PID14 core motif is present in proteins of several plant associated fungi and bacteria, indicating the existence of a conserved microbial inhibitor motif of proteases (Misas Villamil et al., 2019).

In systems not allowing reverse genetics, effectors are often expressed constitutively in plants and effects on SA signaling are then inferred by treating such plants with SA and observing changes in *PR-1* expression. With such an approach, the effector HaRxL44 from the oomycete *Hyaloperonospora arabidopsidis (Hpa)* was shown to suppress *PR-1* expression after SA treatment (Caillaud et al., 2013). HaRxL44 is a nuclear effector interacting with and promoting the proteasomal degradation of the Mediator subunit MED19a likely by acting as an adaptor protein for E3 ligases (Figure 1A; Caillaud et al., 2013). After SA treatment, *PR-1* expression was elevated in *Arabidopsis* plants overexpressing MED19a and *med19a* mutants showed reduced *PR-1* transcript levels. In transgenic *Arabidopsis* plants overexpressing HaRxL44, *PR-1* expression was strongly reduced (Caillaud et al., 2013). Suppression of *PR-1* expression by *Hpa* occurred specifically in cells containing haustoria, sites of delivery of RxLR effectors (Caillaud et al., 2013; Whisson et al., 2016). This indicates that *Hpa* colonization requires HaRxL44-induced destabilization of MED19a to decrease SA-mediated defense responses. Another *Hpa* effector, HaRxL106, was also recently shown to dampen SA-mediated defenses. HaRxL106-expressing lines displayed significantly reduced *PR-1* expression compared to wild type plants after SA treatment (Wirthmueller et al., 2018). *PR-1* expression in *Arabidopsis* plants overexpressing NPR1 was suppressed by HaRxL106, even though neither protein levels nor subcellular localization of NPR1 were affected. Intriguingly, HaRxL106 interacted with radical-induced cell death 1 (RCD1), a nuclear protein shown to activate SA-mediated *PR-1* gene expression (Figure 1A; Wirthmueller et al., 2018). In addition, RCD1 interacted with MUT9-like kinases (MLKs), which phosphorylate photoreceptor cryptochrome 2 (CRY2), phytochrome interacting factor 3 (PIF3) and histone H3 Thr3 (H3T3ph) (Wang et al., 2015; Liu et al., 2017; Ni et al., 2017). The authors speculate that HaRxL106 might act downstream of NPR1 and influence the transcriptional activity of the RCD1/MLK complex to prevent the activation of SA signaling (Wirthmueller et al., 2018). Three additional RxLR effector proteins HaRxL62, HaRxL96 and PsAvh163 from *Hpa* and *P. sojae*, respectively, were also able to suppress SA mediate defenses (Figure 1A; Anderson et al., 2012; Asai et al., 2014). Transgenic *Arabidopsis* plants expressing these effectors individually showed elevated susceptibility to *Hpa* compared to wild type plants. In addition, when challenged with SA, the transgenic plants exhibited reduced *PR-1* expression, suggesting that these effectors compromise SA-triggered immunity (Anderson et al., 2012; Asai et al., 2014). In most of these cases, it remains open at which level *PR-1* gene expression is affected, and further investigations are needed to elucidate the molecular targets of these effectors.

Collectively, these studies demonstrate that filamentous pathogens produce a cocktail of effectors not only to directly disrupt SA homeostasis but also to target more selectively diverse components like NPR1, PLCPs, and PR-1 in the SA signaling pathway. The deployment of effectors for SA pathway interference in fungi and oomycetes indicates convergent evolution to target this important hormone pathway.

## Effectors Targeting the Jasmonic Acid Pathway

The JA pathway has long been thought to allow plants to cope with various environmental stresses including attack by necrotrophic pathogens and herbivores (Thomma et al., 1998, 1999; Glazebrook, 2005). More recently, it has also been shown that JA-mediated defenses contribute to resistance against some biotrophic or hemibiotrophic pathogens (Thaler et al., 2004; Riemann et al., 2013; Lemarie et al., 2015). In rice, JA-mediated defenses conferred immunity against the hemibiotrophic rice blast fungus *Magnaporthe oryzae* (Riemann et al., 2013). To overcome JA-mediated defenses, *M. oryzae* secretes the hydroxylated JA molecule 12OH-JA during the initial biotrophic stage (Patkar et al., 2015). The conversion of JA to 12OH-JA is catalyzed by a secreted fungal monooxygenase Abm (Figure 1B; Miersch et al., 2008; Patkar et al., 2015). It is likely that *M. oryzae* employs Abm to convert both fungal and host-derived JA to 12OH-JA to avoid triggering host JA-mediated immunity (Patkar et al., 2015). Indeed, an *abm* mutant of *M. oryzae* failed to produce blast symptoms on rice and accumulated large amounts of methyl JA (MeJA) in infected tissue, which provoked strong host defense responses (Patkar et al., 2015). This shows that 12OH-JA acts as a metabolite effector blocking JA-triggered defense responses.

By contrast, the hemibiotrophic ascomycete fungus *Fusarium oxysporum* causing root wilt is reported to produce JAs (Cole et al., 2014). JA is precursor of JA-isoleucine (JA-Ile), the ligand of the F-box protein coronatine insensitive 1-jasmonate ZIM-domain (COI1-JAZ) co-receptor complex (Yan et al., 2009). *A. thaliana coi1* plants that are defective in JA signaling exhibited higher resistance against *F. oxysporum* than wild-type plants, indicating that *F. oxysporum* requires COI1-mediated JA signaling to promote virulence (Thatcher et al., 2009). Surprisingly, plant endogenous JA biosynthesis appeared dispensable for COI1-mediated JA signaling during *F. oxysporum* colonization (Thatcher et al., 2009; Cole et al., 2014), making it likely that JA molecules produced by *F. oxysporum* are used in place of plant JA to activate JA signaling (Cole et al., 2014). In addition, it has also been demonstrated that the virulence-promoting secreted in xylem (SIX) effector Fo5176-SIX4 activates JA signaling (Figure 1B; Thatcher et al., 2012). Whether this is direct and at which stage this occurs remain to be determined. The necrotrophic grapevine pathogen *Lasiodiplodia mediterranea* also activates JA signaling by producing the JA ester lasiojasmonate A (LasA) (Figure 1B; Chini et al., 2018). LasA can be converted to JA-Ile, a strong activator of JA signaling and inducer of cell death. LasA is thus proposed to act as a metabolite effector in late stages of infection to activate JA-mediated cell death and facilitate necrotrophy (Chini et al., 2018).

## Effectors Targeting the Ethylene Pathway

The gaseous phytohormone ET is well known for its role in fruit ripening and plant senescence (Burg and Burg, 1965; Grbić and Bleecker, 1995; Bleecker and Kende, 2000). ET-insensitive *A. thaliana* and soybean plants are more susceptible to some pathogens and activation of ET signaling confers plant resistance upon pathogen attack, suggesting that ET signaling also plays a role in plant defense (Hoffman et al., 1999; Thomma et al., 1999; Berrocal-Lobo et al., 2002; Yang et al., 2017b).

The precursor for ET biosynthesis is 1-aminocyclopropane-1-carboxylic acid (ACC), which is derived from S-adenosylmethionine (S-AdoMet) in a reaction catalyzed by ACC synthase (ACS) (Figure 1C; Wang et al., 2002). ET production is directly correlated with ACS activity (Christians et al., 2009; Skottke et al., 2011; Li et al., 2012; Helliwell et al., 2016). The polymorphic RxLR effector PsAvh238 of *P. sojae* is strongly upregulated during early infection and essential for virulence (Figure 1C; Wang et al., 2011b; Yang et al., 2017a). A recent study uncovered that PsAvh238 interacts with soybean Type2 ACSs (GmACSs). By destabilizing GmACSs, PsAvh238 suppresses ET biosynthesis and this facilitates *P. sojae* infection (Figure 1C; Yang et al., 2018a). Silencing of GmACSs as well as inhibition of ET signaling or synthesis with chemical antagonists increased virulence of *P. sojae*, whereas overexpression of GmACSs in *Nicotiana benthamiana* leaves enhanced resistance (Yang et al., 2018a). A *PsAvh238* mutant was unable to inhibit ET signaling and showed reduced virulence (Yang et al., 2018a), consistent with the notion that ET-mediated defenses have to be downregulated in this hemibiotrophic pathosystem.

Conversely, the necrotrophic fungal pathogen *Cochliobolus miyabeanus* causing brown spot of rice requires ET signaling for pathogenesis (Van Bockhaven et al., 2015). While exogenous application of ethephon, which is quickly converted to ET *in planta*, promoted disease development, ET-insensitive rice plants were more resistant to *C. miyabeanus* (De Vleesschauwer et al., 2010). Furthermore, *C. miyabeanus* was shown to produce ET, and the fungus-derived ET constituted the prevalent source of ET in infected tissues (Van Bockhaven et al., 2015). Blocking fungal ET synthesis with specific chemical inhibitors significantly compromised *C. miyabeanus* colonization of rice leaves (Van Bockhaven et al., 2015). This makes it likely that *C. miyabeanus* uses ET as a metabolite effector to promote virulence (Van Bockhaven et al., 2015).

## Effectors Targeting the Auxin Pathway

Auxins constitute a group of indolic molecules that have long been recognized for their multiple roles in plant growth, development, and pathogen-host interactions (Teale et al., 2006; Barbier et al., 2017). *A. thaliana* mutants defective in auxin signaling were more susceptible to the necrotrophic fungi *Plectosphaerella cucumerina* and *B. cinerea*, and the application of the auxin transport inhibitor 2,3,5-triiodobenzoic acid (TIBA) rendered *Arabidopsis* more susceptible to *P. cucumerina* (Llorente et al., 2008).

Several microorganisms induce plant galls/tumors with high auxin levels. These structures likely provide the environment for pathogen differentiation and/or pathogen dissemination (Yamada, 1993; Kazan and Manners, 2009). One such example where tumors were shown to contain high IAA (indole-3-acetic acid) auxin levels are those induced by *U. maydis* (Turian and Hamilton, 1960). *U. maydis* is able to produce IAA from tryptophan (Reineke et al., 2008). Deleting two IAA dehydrogenases and a transaminase genes resulted in significantly reduced IAA production of *U. maydis* in axenic culture, but tumor formation was unaltered and IAA levels in in tumors were indistinguishable from those in wild type infections (Reineke et al., 2008). This suggested that fungal IAA does neither act as effector for tumor induction nor for elevating IAA levels in tumor tissue (Reineke et al., 2008).

The stem rust fungus *Puccinia graminis* f. sp. *tritici (Pgt)* produces a putative tryptophan 2-monooxygenase (Pgt-IaaM) that generates indole-3-acetamide (IAM), the precursor for IAA biosynthesis (Yin et al., 2014). In *Pgt*-infected wheat leaves, the expression of *Pgt-IaaM* was strongly induced in haustorial cells, and higher IAA levels were observed. Silencing of *Pgt-IaaM* during *Pgt* infection on wheat *via* host-induced gene silencing (HIGS) compromised the virulence of *Pgt*, whereas transgenic *Arabidopsis* plants constitutively expressing Pgt-IaaM displayed increased accumulation of IAA and susceptibility to biotic stress. This suggests that *Pgt* produced IAA acts as a virulence promoting effector in this pathosystem (Yin et al., 2014).

Directional auxin transport is controlled by an efflux carrier complex containing the PIN-formed (PIN) family proteins (Robert and Friml, 2009). The RxLR effector penetration-specific effector 1 (PSE1) of the oomycete *Phytophthora parasitica* infecting *Arabidopsis* is transiently upregulated during penetration of host roots (Evangelisti et al., 2013). The overexpression of PSE1 in *Arabidopsis* reduced auxin accumulation and increased susceptibility to *P. parasitica*. In addition, PSE1 expressing *Arabidopsis* plants displayed significantly enhanced accumulation of auxin exporters PIN4 and PIN7 at the root apex (Figure 1D; Evangelisti et al., 2013). It is therefore proposed that PSE1 promotes infection by altering auxin physiology (Evangelisti et al., 2013). How PSE1 achieves this mechanistically remains to be determined.

## Effectors Targeting the Brassinosteroid Pathway

Brassinosteroids (BRs) are a class of polyhydroxylated steroidal phytohormones that are implicated in a wide range of plant physiological and developmental processes as well as plant defense responses (Clouse, 2011; Wang et al., 2012). It was shown that activation of BR signaling increases the susceptibility of *A. thaliana* and rice to *Hpa* and *Pythium graminicola*, respectively (Belkhadir et al., 2012; De Vleesschauwer et al., 2012). BR insensitive 1 (BRI1) is a leucine-rich repeat-receptor-like kinase (LRR-RLK), which perceives and transduces BR signals (Wang et al., 2001). BR signaling also involves the Kelch-repeat containing protein phosphatase BRI1 suppressor 1 (BSU1) (Mora-García et al., 2004). In *A. thaliana*, BR signaling regulates the tradeoff between plant growth and defense *via* modulating the transcription factors brassinazole-resistant 1 (BZR1) and homolog of brassinosteroid enhanced expression2 interacting with ibh1 (HBI1), two negative regulators of plant defense responses (Lozano-Durán and Zipfel, 2015). The oomycete *P. infestans* secretes the RxLR effector AVR2 during its biotrophic stage of potato colonization (Figure 1E; Gilroy et al., 2011). AVR2 interacts with the phosphatase BSU-like protein1 (BSL1), which is homologous to *A. thaliana* BSU1, suggesting a possible link between AVR2 and BR signaling (Saunders et al., 2012). Indeed, overexpression of AVR2 in potato constitutively activated the expression of several BR responsive genes including *StCHL1*, a basic helix-loop-helix (bHLH) transcription factor homologous to HBI1 in *A. thaliana* (Figure 1E; Turnbull et al., 2017). Transient overexpression of either AVR2 or StCHL1 facilitated disease development of *P. infestans* on *N. benthamiana*, whereas silencing of the *CHL1* ortholog of *N. benthamiana* compromised susceptibility to *P. infestans* (Turnbull et al., 2017). It is hypothesized that AVR2 hijacks StCHL1 to activate BR signaling and suppress plant immunity (Turnbull et al., 2017). This highlights a new strategy to suppress plant immunity *via* exploiting the tradeoff between plant growth and immunity.

## Effectors Targeting the Cytokinin Pathway

Cytokinins (CKs) are N^6^-substituted adenine derivatives playing important roles in plant development and stress responses (Kieber and Schaller, 2018). The CK biosynthetic pathway involves adenylate isopentenyltransferase (IPT), cytochrome P450 monooxygenase and LONELY GUY (LOG) enzymes (Takei et al., 2001, 2004; Sakakibara, 2006; Kurakawa et al., 2007). CKs can also originate from degradation of modified tRNAs in a reaction catalyzed by tRNA-IPT (Miyawaki et al., 2006). CK biosynthetic genes are detected in many plant pathogens, suggesting that CKs might be produced by pathogens to promote disease (Chanclud and Morel, 2016; Sorensen et al., 2018).

The ergot fungus *Claviceps purpurea* produces large amount of CKs in axenic culture and during early infection. The deletion of *CptRNA-IPT* abolished the production of *cis*-zeatin (*c*Z) and reduced virulence while mutants lacking either a bifunctional *CpIPT-LOG* or *CpP450* were unaffected in virulence (Hinsch et al., 2015, 2016). However, double mutants lacking *CpIPT-LOG* and *CptRNA-IPT* were severely attenuated in virulence, illustrating that both fungal CK production pathways contribute to virulence in this pathosystem (Hinsch et al., 2016). *U. maydis* also produces *c*Z-type CKs in axenic culture and in infected plant tissues (Bruce et al., 2011; Morrison et al., 2015). *U. maydis* mutants lacking *tRNA-IPT* were deficient in *c*Z synthesis and showed reduced virulence in seedling infection in comparison to wild type strains (Morrison et al., 2017). Similarly, the deletion of *CKS1* encoding tRNA-IPT in *M. oryzae* also led to a significant reduction of rice blast symptoms and increased plant defense responses (Chanclud et al., 2016). The virulence defect of the *CKS1* mutant could be restored when CK was exogenously applied, reinforcing the link between CK production and pathogenicity (Chanclud et al., 2016). Recently, a new class of cytokinins called Fusarium cytokinins was shown to be produced by the cereal pathogen *Fusarium pseudograminearum* (Sorensen et al., 2018). Fusarium cytokinins acts as cytokinin agonists and could activate a histidine kinase 3 cytokinin receptor in a heterologous bacterial system. The expression of the biosynthetic gene cluster for Fusarium cytokinins was induced during *F. pseudograminearum* infection of barley, but a role in virulence has not yet been demonstrated (Sorensen et al., 2018).

It is thus likely that in these fungal pathosystems CKs function as effectors to suppress host immune responses during colonization.

## Effectors Targeting the Gibberellin Pathway

Gibberellins (GAs) were initially identified from *Fusarium fujikuroi*, which is causative for “bakanae” disease of rice seedlings (Wulff et al., 2010). Later, GAs were also found to be produced by plants and shown to play vital roles in regulating plant growth and development (Yamaguchi, 2008). GA biosynthetic genes of *F. fujikuroi* are strongly induced in rice roots colonized by *F. fujikuroi*. In comparison to the GA-producing wild type strain, GA-nonproducing mutants lacking the entire GA biosynthetic gene cluster showed comparable root penetration and apoplastic growth but were compromised in further invasion of rice tissue, suggesting that secreted GA is used as effector for “bakanae” disease development (Wiemann et al., 2013). However, the GA biosynthetic gene cluster is restricted to *Fusarium* species but GA production is only found in *F. fujikuroi* (Wiemann et al., 2013). It remains unclear how GA contributes mechanistically to the virulence of *F. fujikuroi*. In the *M. oryzae*/rice pathosystem, it has been demonstrated that mutants lacking GA inactivating enzyme elongated uppermost internode (EUI) were more susceptible to infection by *M. oryzae*, whereas mutants in gibberellin 20-oxidase (GA20OX3) involved in GA synthesis showed increased rice blast resistance. This indicates that the GA pathway also plays a positive role in this pathosystem (Yang et al., 2008; Qin et al., 2013).

## Effectors Targeting the Abscisic Acid Pathway

Abscisic acid (ABA) is a sesquiterpenoid synthesized *via* two distinct pathways involving the proteins ABA1, ABA4, NCED, ABA2, and ABA3 (Endo et al., 2014). It is an essential hormone regulating plant developmental processes and adaptive responses to diverse abiotic and biotic stresses (Cutler et al., 2010). ABA has a negative role on plant resistance against some biotrophic filamentous pathogens, such as *Hpa, Fusarium graminearum, M. oryzae*, and *Golovinomyces cichoracearum* (Fan et al., 2009; Jiang et al., 2010; Buhrow et al., 2016; Xiao et al., 2017). On the other hand, plants require the ABA pathway for resistance against several necrotrophic pathogens, including *Pythium irregulare, P. cucumerina, C. miyabeanus*, and *Alternaria brassicicola* (Ton and Mauch-Mani, 2004; Adie et al., 2007; Fan et al., 2009; De Vleesschauwer et al., 2010). Fungi like *M. oryzae, U. maydis*, and *B. cinerea* are able to synthesize ABA (Siewers et al., 2006; Bruce et al., 2011; Spence et al., 2015). An *aba4* mutant of *M. oryzae* lacking one of the biosynthetic genes for ABA biosynthesis and producing reduced ABA levels was severely affected in appressoria formation on a hydrophobic surface in comparison to the wild type strain. Exogenous ABA application largely restored this defect (Spence et al., 2015). Furthermore, the *aba4* mutant lost the ability to infect rice. However, the *aba4* mutant also displayed a strong growth defect and morphological abnormalities which could not be complemented by external ABA (Spence et al., 2015). So far, it has not been possible to separate the endogenous function from a function of ABA as virulence-promoting effector in this system.

## Effectors Targeting Phytohormone Crosstalk

Hormonal signaling pathways are often interconnected, and this can lead to synergistic or antagonistic functions (Weiss and Ori, 2007; Choi et al., 2010; Jiang et al., 2010; Argueso et al., 2012; Pieterse et al., 2012; Naseem et al., 2014, 2015; Berens et al., 2017). Examples are the antagonism between SA and JA pathway (Kunkel and Brooks, 2002; Takahashi et al., 2004; Spoel and Dong, 2008), and the synergism between JA and ET pathways (Xu et al., 1994; Lorenzo et al., 2003; Zhu et al., 2011) in defense signaling. Furthermore, growth-promoting hormones rely on crosstalk with defense-related hormones to balance growth-defense tradeoffs (Pieterse et al., 2009; Huot et al., 2014; Berens et al., 2017). In this context, fungal-derived phytohormones can be effectors directly influencing the crosstalk with other hormones. Interference with phytohormone crosstalk can also involve polysaccharide: the necrotrophic fungus *B. cinerea* secretes the exopolysaccharide (EPS) β-(1,3)(1,6)-D-glucan to promote infection of tomato (Figure 1B; Stahmann et al., 1995; El Oirdi et al., 2011). Tomato leaves pretreated with EPS were more susceptible to *B. cinerea* and showed enhanced SA accumulation but decreased expression of JA-marker genes *PI I* and *PI II* (El Oirdi et al., 2011). The expression of NPR1 was also induced after *B. cinerea* infection and knockdown of *NPR1* led to significantly increased expression of these two JA-marker genes. This suggests that EPS functions as an effector which activates SA signaling and inhibit the JA signaling pathway *via* NPR1 (Figure 1B; El Oirdi et al., 2011).

## Conclusions and Outlook

Studies in recent years have uncovered that most filamentous plant pathogens use interference with hormonal pathways as an effective strategy to promote colonization. Nearly, all phytohormone pathways can be targeted with beneficial consequences for the pathogens. The mechanisms how filamentous plant pathogens induce changes in phytohormone levels and/or signaling have become more complex by realizing that many filamentous phytopathogens can also produce phytohormones or derivatives for host phytohormone mimicry. The regulation of hormone production and deployment by fungal plant pathogens is only beginning to surface and indicates that phytohormones can be used as effectors during plant colonization. It is currently unknown why phytohormone effectors have so far not been detected in oomycete pathogens, which are phylogenetically related to brown algae but distinct from fungal lineages.

Currently, we do not understand why some pathogens use protein effectors to target hormone biosynthesis or signaling, while others shift hormonal balances by producing hormones or hormone mimics. It is at least conceivable that the use of microbial hormones as virulence factors may make it difficult for the plant to mount defense responses as these would also target the respective endogenous plant pathways. This could suggest that phytohormone effectors might be rather new additions to the battle between phytopathogens and their hosts. A comprehensive study on the evolution of such microbial traits will be needed to settle this point in future.

Given the large effector repertoire of filamentous pathogens, it is only a small number of effectors that target phytohormone pathways. Currently, it appears that the SA pathway is most extensively exploited by filamentous plant pathogens. We consider that this might be due to the fact that simple and sensitive readouts such as cell death or *PR-1* expression have been developed that provide an easy way to visualize even subtle effects. Presently, only a small number of effectors have been shown to target other phytohormone pathways and for strigolactone signaling so far no modulating pathogen effectors have been detected. To uncover pathogen effectors which affect these pathways, it might be helpful to develop biosensors using promoter-fluorescent protein fusions for the activity of these pathways and employ such biosensors in high-throughput effectoromic studies to uncover effectors up or downregulating these reporter genes. This would also allow to uncover effectors with redundant functions in phytohormone signaling. With the advent of CRISPR-Cas9 technologies in filamentous pathogens, it should then become feasible to relate such redundant effectors with virulence. The fast-growing progress in multi-omics of filamentous plant pathogens and techniques for identifying protein interactions will further accelerate the discovery of filamentous pathogen effector targets and the underlying mechanism for manipulating phytohormone pathways.

Given the prominent involvement of phytohormones in defense, it is not surprising that engineering phytohormone pathways has potential for field applications. However, the opposing effects of phytohormones on disease caused by filamentous pathogens with different life styles do not allow to follow a straightforward strategy. Therefore, rather than simply increasing or decreasing hormone levels, it may be safer to modify effector targets by plant genome editing approaches like TILLING or CRISPR-Cas9 so that they can evade effector interference. This might also allow to get around the problem that higher levels of certain growth-related hormones will kick-off a trade-off between growth and defense. In addition, for real field situations, it needs to be kept in mind that plants are associated with a complex microbiome. So far, we largely lack studies that address the importance of phytohormone signaling pathways with respect to defense when plants growing in the field receive a cocktail of different signals. Evidence is accumulating that the innate immune system of plants as a whole serves both pathogen elimination and controlled accommodation of beneficial microbes (Hacquard et al., 2017). This will make it necessary to explore the role of phytohormones in plant-microbe interactions in the context of filamentous plant pathogens living in more complex natural environments.

## Author Contributions

All authors listed have made a substantial, direct and intellectual contribution to the work, and approved it for publication.

### Conflict of Interest Statement

The authors declare that the research was conducted in the absence of any commercial or financial relationships that could be construed as a potential conflict of interest.
